# Blood pressure and cardiovascular diseases in Chinese adults with type 2 diabetes: A prospective cohort study

**DOI:** 10.1016/j.lanwpc.2020.100085

**Published:** 2021-01-23

**Authors:** Fiona Bragg, Jim Halsey, Yu Guo, Hua Zhang, Ling Yang, Xiaohui Sun, Pei Pei, Yiping Chen, Huaidong Du, Canqing Yu, Robert Clarke, Jun Lv, Junshi Chen, Liming Li, Zhengming Chen, for the China Kadoorie Biobank (CKB) collaborative group (members listed in Supplementary appendix)

**Affiliations:** a Clinical Trial Service Unit & Epidemiological Studies Unit (CTSU), Nuffield Department of Population Health, University of Oxford, Old Road Campus, Oxford OX3 7LF, UK; b Medical Research Council Population Health Research Unit at the University of Oxford, Oxford, UK; c Chinese Academy of Medical Sciences, Beijing 102308, China; d Qingdao Center for Disease Control and Prevention, 175 Shandong Road, Qingdao 266033, China; e School of Public Health, Peking University Health Science Center, Beijing, China; f China National Center For Food Safety Risk Assessment, Beijing 100022, China

**Keywords:** Blood pressure, Cardiovascular disease, China, Diabetes, Hypertension, Ischaemic heart disease, Stroke

## Abstract

**Background:**

Controversy persists about the relationship of blood pressure with cardiovascular diseases (CVD) in diabetes and associated disease burden. We assessed these associations among Chinese adults with type 2 diabetes (T2D).

**Methods:**

In 2004–08, the China Kadoorie Biobank recruited >512,000 adults aged 30–79 years from 10 localities across China, including 26,315 with T2D (based on self-report or plasma glucose measurement) but no prior CVD, followed-up for ~9 years. Cox regression yielded adjusted HR for major CVD and all-cause mortality associated with 10 mmHg higher usual (longer-term average) SBP. Attributable fractions were estimated to assess cardiovascular mortality burden due to uncontrolled hypertension (SBP ≥130 mmHg or DBP ≥80 mmHg).

**Findings:**

Overall, 75.7% of participants had self-reported (24.8%) or screen-detected (50.9%) (SBP ≥130 mmHg or DBP ≥80 mmHg) hypertension. Among individuals with self-reported hypertension, 82.3% were treated, of whom 9.3% achieved control. There were positive log-linear associations of blood pressure with CVD, with no evidence of a threshold down to ~120 mmHg for usual SBP. Each 10 mmHg higher usual SBP was associated with HR of 1.28 (95% CI 1.25–1.30), 1.18 (1.15–1.21), 1.17 (1.15–1.19) and 1.45 (1.38–1.52) for cardiovascular death (*n*=1807), major coronary event (*n*=1190), ischaemic stroke (*n*=4362) and intracerebral haemorrhage (*n*=469), respectively. There was an apparent J-shaped association with all-cause mortality (*n*=4503). In this diabetes population, uncontrolled hypertension accounted for 39% of cardiovascular deaths.

**Interpretation:**

Uncontrolled hypertension is common in Chinese adults with T2D, resulting in substantial excess risks of CVD. Improved hypertension management could avoid a large number of cardiovascular-related deaths.

**Funding:**

Kadoorie Foundation, Wellcome Trust, MRC, BHF, CR-UK, MoST, NNSF.

**Research in context**

Evidence before this study

Despite clear evidence of lower cardiovascular disease risk with intensive control of blood pressure in the general population, uncertainty persists regarding blood pressure targets in diabetes, and most existing evidence is derived from Western population studies. We searched PubMed for articles published up to August 2020 using the terms “blood pressure” (or “hypertension”) and “cardiovascular disease” (or “ischaemic heart disease” or “stroke”) and “diabetes” and “China” (or “Chinese”). Reference lists of identified relevant articles were also reviewed. A small number of prospective studies were identified based in mainland China and Hong Kong. These studies all reported positive associations of blood pressure with risks of cardiovascular diseases. However, most studies were small and did not account for intra-individual variation in blood pressure, and none examined the associations of blood pressure with stroke types. Moreover, although previous studies in China have estimated the prevalence and control of hypertension among adults with diabetes, none has combined these estimates with associated cardiovascular disease risks, and the burden of cardiovascular diseases associated with hypertension in this population remains unclear.

Added value of this study

This prospective study aimed to quantify the association of blood pressure with major cardiovascular diseases, and estimate the cardiovascular mortality burden associated with uncontrolled hypertension among Chinese adults with type 2 diabetes. Among the 26,315 participants with type 2 diabetes but no prior cardiovascular diseases, 75.7% had hypertension. Just one third of individuals with hypertension had been previously diagnosed, of whom approximately 80% were treated. Despite the high frequency of treatment, overall, 67.1% of adults with type 2 diabetes had uncontrolled hypertension. Higher blood pressure was associated with higher risks of cardiovascular disease and its main types, with no evidence of a threshold in the associations down to a usual (longer-term average) systolic blood pressure of at least ~120 mmHg, providing support for more intensive blood pressure treatment targets in diabetes. Each 10 mmHg higher usual systolic blood pressure was associated with approximately 20% higher risks of ischaemic cardiovascular diseases (major coronary event and ischaemic stroke), and 45% higher risk of intracerebral haemorrhage. The association with cardiovascular death was intermediate between these. Overall, uncontrolled hypertension accounted for 39% of cardiovascular deaths in this population.

Implications of all the available evidence

Diabetes prevalence is high and increasing worldwide. Cardiovascular diseases remain the most common cause of death in this population, in part reflecting clustering of diabetes with other cardiovascular risk factors, including elevated blood pressure. Hypertension is common, underdiagnosed and poorly controlled among Chinese adults with diabetes. Moreover, there are strong, continuous, positive associations of blood pressure with the risk of cardiovascular diseases. Uncontrolled hypertension results in substantial unnecessary morbidity and mortality in this population, and improved management of hypertension among individuals with type 2 diabetes would be expected to avoid a large number of cardiovascular-related deaths in China.

Alt-text: Unlabelled box

## 1 Introduction

Cardiovascular diseases (CVD) are the most common causes of death in diabetes [1], partly reflecting frequent clustering with adiposity, elevated blood pressure and other CVD risk factors [2]. However, controversy persists regarding the relationships between blood pressure and risk of CVD among people with diabetes, and the associated disease burden.

Large prospective observational studies [3,4] and randomised controlled trials [5] have consistently demonstrated that intensive control of blood pressure (target systolic blood pressure [SBP] <120 mmHg) is associated with lower risks of CVD in the general population. However, the evidence is less clear among individuals with diabetes, with some studies reporting comparable risks to those in the general population [3,^6^,7], while others have reported higher CVD [8,9] and all-cause [8] mortality risks at lower SBP levels (SBP <130 mmHg [8] or <120 mmHg [9]). Furthermore, the large ACCORD BP trial failed to demonstrate any benefit of a SBP target of <120 mmHg when compared with <140 mmHg among individuals with type 2 diabetes (T2D) [10], with meta-analyses of trial data suggesting possible adverse CVD [11] and non-CVD [12] effects of intensive blood pressure lowering. These uncertainties are reflected in treatment guidelines, variably recommending 130/80 mmHg [13–15] and 140/90 mmHg [16] as cut-offs for initiation of anti-hypertensive treatment or treatment targets in diabetes.

Most previous large studies of the association of blood pressure with CVD in diabetes involved mainly Western populations [6,^8^,9]. Hypertension and diabetes are more common and frequently less well-controlled in China [17], [18], [19]], and population differences are proposed in the underlying pathophysiology of T2D [20]. Evidence from Western population studies may not be generalisable and reliable evidence is required in East Asian populations, particularly in China with the largest diabetes population globally [1]. Moreover, patterns and burden of CVD differ in China from those in Western populations, with typically higher rates of stroke, particularly haemorrhagic stroke [21], providing a unique opportunity to study risks and determinants of stroke and stroke types in diabetes.

We examine the burden of hypertension, and the associations of blood pressure with major CVD incidence and mortality among Chinese adults with T2D, and estimate the burden of CVD associated with uncontrolled hypertension.

## 2 Methods

### 2.1 Study population

The China Kadoorie Biobank (CKB) design, methods and population have been previously described [22]. Briefly, permanent, non-disabled residents aged 35–74 years from 100 to 150 rural villages or urban committees in each of 10 study areas (5 urban and 5 rural) were invited to participate. Study areas were selected from China's nationally representative Disease Surveillance Points (DSP) to ensure diversity in socioeconomic levels and patterns of risk factors and disease. The overall response rate was ~30%, and 512,713 men and women were recruited, including 13,215 slightly outside the target age range. Ethics approval was obtained from relevant local, national and international ethics committees prior to commencement of the study. All participants provided informed written consent.

### 2.2 Data collection

During the 2004–08 baseline survey, trained health workers administered laptop-based questionnaires, collecting information on socio-demographics, lifestyle, and medical history. Among individuals with self-reported prior IHD, stroke, hypertension or diabetes, data were collected on current use of cardiovascular medications. Physical measurements were undertaken using calibrated instruments and standard protocols. A non-fasting venous blood sample was collected and time since last food recorded. Plasma glucose levels were measured immediately using the SureStep Plus meter (LifeScan, Milpitas, CA). Participants with a plasma glucose level ≥7.8 mmol/L and <11.1 mmol/L were invited to return the following day for fasting plasma glucose measurement. Resurveys were undertaken in 2008 and 2013–14, including a 5% randomly-selected sample of surviving participants and collating the same information as the baseline survey.

### 2.3 Assessment of blood pressure and hypertension status

Blood pressure was measured twice using a UA-779 digital sphygmomanometer (A&D Instruments; Abingdon, UK) after at least five minutes resting in a seated position. A third blood pressure measurement was taken if the difference between the two SBP measurements was >10 mmHg. The last two readings were recorded, and their mean used. Self-reported hypertension was defined as a “yes” response to the question “Has a doctor ever told you that you have hypertension?” Screen-detected hypertension was defined as no self-reported hypertension and a mean measured SBP ≥130 mmHg or diastolic blood pressure (DBP) ≥80 mmHg [13]. Uncontrolled hypertension was defined as measured SBP ≥130 mmHg or DBP ≥80 mmHg [13]. Awareness of hypertension was the proportion of all hypertension that was self-reported.

### 2.4 Definition of type 2 diabetes

Diabetes was defined as self-reported doctor-diagnosed (*n*=16,162) or screen-detected (no self-reported diabetes and plasma glucose ≥7.0 mmol/L and fasting time ≥8 h, plasma glucose ≥11.1 mmol/L and fasting time <8 h, or subsequent fasting plasma glucose ≥7.0 mmol/L) (*n*=14,137) diabetes at baseline [23]. T2D excluded individuals who were <35 years at diagnosis and reported taking insulin (*n*=102).

### 2.5 Follow-up for morbidity and mortality

Participants were followed-up for cause-specific morbidity and mortality through linkage, via unique national identification numbers, to death registries at China's DSP (ICD-10 coded by trained staff blinded to baseline information), to disease registries for diabetes, stroke, IHD and cancer, and to the national health insurance system (providing details of ICD-10 coded diagnoses resulting in or during hospitalisations).

To minimise loss to follow-up and under-reporting of deaths, active follow-up was undertaken annually by checking local residential and administrative records, and, if necessary, by contacting participants’ family members. By 1 January 2017, there were 42,922 deaths, including 5692 among participants with T2D, and <1% of participants were lost to follow-up.

The CVD endpoints examined in the present study included cardiovascular death (ICD-10 I00–I25, I27–I88, I95–I99), major coronary event (MCE) (non-fatal myocardial infarction [ICD-10 I21–I23] or fatal IHD [ICD-10 I20–I25]), ischaemic stroke (IS) (ICD-10 I63), intracerebral haemorrhage (ICH) (ICD-10 I61) and total stroke (ICD-10 I60, I61, I63, I64).

### 2.6 Statistical analysis

The main analyses, among participants with T2D, excluded, sequentially, individuals with self-reported doctor-diagnosed IHD, stroke or transient ischaemic attack (TIA) (*n*=3872), or extreme or improbable SBP (<80 or ≥250 mmHg, *n*=8) or DBP (<40 or ≥150 mmHg, *n*=2). After these exclusions, the remaining 26,315 participants with T2D were included in the main analyses. After excluding individuals based on the same criteria (self-reported IHD, stroke or TIA *n*=19,244; extreme/improbable SBP *n*=94; extreme/improbable DBP *n*=43) or with missing body mass index (BMI) data (*n*=2), we examined risk associations separately among 463,031 participants without diabetes for comparison.

Mean values and prevalence of baseline characteristics were calculated across ACC/AHA defined hypertension categories [13]. Prevalence, treatment and control of hypertension were assessed across categories of sociodemographic characteristics, standardised by 5-year age group, sex and study area, overall and separately among participants with self-reported and screen-detected diabetes.

Cox proportional hazards models were used to estimate hazard ratios (HRs) for the associations of hypertension (normotensive [reference]/elevated blood pressure/stage 1 hypertension/stage 2 hypertension/hypertensive crisis) [13] and, separately, uncontrolled hypertension, with incident CVD and all-cause mortality, with time since entry into the study as the underlying timescale. Models were stratified by age-at-risk, sex and study area, and adjusted for educational attainment, smoking, alcohol consumption, physical activity and BMI. Separate Cox regression analyses, adjusting for the same variables, examined the associations of SBP and DBP, categorised to enable investigation of the full distribution while ensuring adequate CVD events in each category (cut-points of 10th, 20th, 40th, 60th, 80th percentiles). When examining associations of hypertension and blood pressure as categorical variables, group-specific variances were used to calculate 95% CI for all categories (reflecting the amount of data in each individual category), enabling comparisons between any two categories and not only with the reference group [24]. Where associations with outcomes were log-linear, SBP and DBP were examined as continuous variables to estimate HR per 10 mmHg and 5 mmHg higher SBP and DBP, respectively. Follow-up was censored on the date of a CVD event, death, loss to follow-up or at the censoring date (1 January, 2017), whichever occurred first.

Blood pressure measured at baseline may not accurately reflect an individual's longer-term average (or “usual”) blood pressure due to measurement error and short and long-term within-person variation. In prospective studies, failure to account for this may result in so-called regression dilution bias, with substantial under-estimation of the relevance of blood pressure for disease risks [25]. Blood pressure measurements among 948 individuals with T2D from the 2008 resurvey were used to estimate usual SBP and DBP. Log HR estimates for baseline SBP and DBP were multiplied by the reciprocal of the regression dilution ratio (0.62 for both SBP and DBP, estimated as the coefficient for baseline SBP/DBP measurements when regressing resurvey SBP/DBP measurements against baseline measurements [26]) to estimate the strength of the associations of usual SBP and DBP. Adjusted HRs were calculated across strata of other baseline variables (e.g., age, sex, study area, lifestyle factors), and chi-square tests for trend and heterogeneity were applied to log HRs and their SEs.

Further analyses additionally mutually adjusted for SBP/DBP to examine independent effects, examined the associations of SBP and DBP separately among individuals with self-reported and screen-detected T2D, and individuals with and without self-reported hypertension, and, in order to investigate the role of reverse causality, included individuals with prior CVD and, separately, additionally excluded individuals with other chronic diseases and poor self-rated health at baseline. Comparison of HRs for the first four and subsequent years of follow-up revealed no evidence of deviation from the proportional hazards assumption. Estimates of 10-year CVD risks by hypertension and diabetes status were calculated using a multi-variable Poisson regression model adjusted for age, sex, study area, educational attainment, smoking, alcohol consumption, physical activity and BMI, censoring at the first CVD event. The age- and sex-specific proportions of cardiovascular deaths attributable to uncontrolled hypertension were calculated as P*([HR-1]/HR), where *P* is the proportion of individuals with T2D who died due to CVD with uncontrolled hypertension and HR is the adjusted HR for cardiovascular death in participants with vs. without uncontrolled hypertension [27].

All analyses were conducted using SAS version 9.4.

### 2.7 Role of the funding source

The funders of the study had no role in the design of the study, collection, analysis or interpretation of data, or in the writing of the report.

## 3 Results

Among 26,315 participants with T2D but no prior CVD at baseline, the mean (SD) age was 57.4 (9.6) years, 38.7% were men and 58.4% lived in urban areas (Table 1). Ever regular smoking was reported by 72.0% of men and 4.7% of women. Overall, mean (SD) SBP and DBP were 141 (22) mmHg and 80 (11) mmHg, respectively. Individuals with higher blood pressure were more likely to be older, live in rural areas, have lower educational attainment, and higher BMI and waist circumference and, among men, were more likely to regularly drink alcohol (*p* for trend <0.0001 for all comparisons).

**Table 1:** Baseline characteristics of individuals with diabetes by hypertension status.

Characteristic^a^	Normotensive	Elevated blood pressure	Stage 1 hypertension	Stage 2 hypertension	Hypertensive crisis	Total
No. of participants	3837	3111	6158	11,712	1497	26,315
Mean SBP (SD), mmHg	110 (9)	125 (9)	132 (9)	154 (9)	193 (9)	141 (22)
Mean DBP (SD), mmHg	69 (9)	72 (9)	79 (9)	84 (9)	96 (9)	80 (11)
**Age and socioeconomic factors**						
Mean age (SD), years	54.1 (9.3)	56.9 (9.3)	55.9 (9.3)	59.0 (9.3)	61.4 (9.3)	57.4 (9.6)
Men, %	34.7	37.9	41.5	39.3	32.5	38.7
Living in urban area, %	66.3	60.4	58.8	56.3	49.9	58.4
6+ years of education, %	54.2	48.0	46.6	42.7	36.7	45.7
**Lifestyle factors**						
Ever regular smoker, %						
Men	75.6	69.7	72.4	71.2	75.0	72.0
Women	6.6	5.5	5.0	3.8	4.0	4.7
Ever regular alcohol drinker, %						
Men	39.2	40.0	42.5	49.3	52.2	45.3
Women	3.2	2.5	2.6	2.3	3.1	2.6
Mean physical activity (SD), MET-h/day	15.2 (11.3)	16.5 (11.1)	16.2 (11.2)	16.4 (11.2)	16.5 (11.2)	16.2 (12.1)
**Anthropometry, plasma glucose and lipids, mean (SD)**						
BMI, kg/m^2^	23.4 (3.5)	24.3 (3.5)	24.9 (3.5)	25.6 (3.5)	25.7 (3.5)	24.9 (3.6)
Waist circumference, cm	81 (10)	84 (10)	85 (10)	87 (10)	87 (10)	85 (10)
LDL-cholesterol^b^, mmol/L	2.5 (0.8)	2.5 (0.8)	2.6 (0.8)	2.6 (0.8)	2.7 (0.8)	2.6 (0.8)
HDL-cholesterol^b^, mmol/L	1.2 (0.3)	1.2 (0.3)	1.2 (0.3)	1.1 (0.3)	1.2 (0.3)	1.2 (0.3)
Triglycerides^b^, mmol/L	2.4 (2.3)	2.7 (2.3)	2.9 (2.3)	2.8 (2.3)	2.9 (2.3)	2.8 (2.3)
Random plasma glucose^c^, mmol/L	12.0 (5.7)	12.6 (5.6)	12.3 (5.6)	12.2 (5.7)	12.7 (5.7)	12.3 (5.6)
**Medical history and medications, %**						
Prior self-reported diseases						
Hypertension	4.9	10.0	15.1	31.3	52.5	24.8
Chronic kidney disease	2.0	1.7	1.7	1.7	2.0	1.8
Cardiovascular medication use^d^						
Anti-hypertensive						
ACE-inhibitor	1.6	2.5	3.7	6.9	9.8	5.6
Beta-blocker	1.6	2.4	3.2	5.7	10.2	4.9
Calcium channel blocker	3.1	5.9	7.7	12.9	16.2	10.6
Diuretic	0.5	0.8	1.0	1.6	1.9	1.3
Any	5.6	10.6	14.0	23.8	33.4	19.6
Statin	1.1	0.3	0.6	1.2	1.5	1.1
Aspirin	1.0	0.8	1.7	3.3	4.9	2.8
**Family medical history, %**						
IHD	2.9	3.0	2.9	3.2	2.8	3.0
Stroke	15.1	17.3	17.3	18.2	18.5	17.4

a Standardised to age, sex and study area structure of the population, as appropriate.

b Data available for 1557 participants.

c Data available for 26,157 participants.

d Data available for 13,408 participants. Normotensive: SBP <120 mmHg and DBP <80 mmHg; Elevated blood pressure: SBP 120–129 mmHg and DBP <80 mmHg; Stage 1 hypertension: SBP 130–139 mmHg or DBP 80–89 mmHg; Stage 2 hypertension: SBP 140–179 mmHg or DBP 90–119 mmHg; Hypertensive crisis: SBP ≥180 mmHg or DBP ≥120 mmHg; individuals who fall into two hypertension categories are designated to the higher category. BMI=body mass index; DBP=diastolic blood pressure; HDL=high density lipoprotein; IHD=ischaemic heart disease; LDL=low density lipoprotein; MET-h/day=metabolic equivalent of task hours per day; SBP=systolic blood pressure.

### 3.1 Prevalence, treatment and control of hypertension

Overall, 75.7% of participants had self-reported (24.8%) or screen-detected (50.9%) hypertension (Table 2). Awareness of hypertension was greater at older ages, and among women and urban residents, but was consistently <40%. Among individuals with self-reported hypertension, 82.3% were treated; the proportion was modestly higher at older ages, and among women and urban residents, and the majority (86.4%) were treated with a single anti-hypertensive agent. Calcium channel blockers were the most commonly used anti-hypertensive agents (23.8%), with just 12.7% taking ACE-inhibitors. Blood pressure control (<130/80 mmHg [13]) was achieved among 9.3% of those on treatment, and overall 67.1% of individuals with T2D had uncontrolled hypertension. Hypertension awareness was lower in screen-detected, than self-reported, T2D (23.9% vs. 41.5%) (**Table S1**). Using alternative SBP and DBP cut-points of 140 mmHg and 90 mmHg, respectively, for hypertension diagnosis [28] and control [16], 31.3% had screen-detected hypertension, and 20.2% of self-reported hypertension was controlled (**Table S2**).

**Table 2:** Prevalence, treatment and control of hypertension among individuals with diabetes

Characteristic	No. of participants	Self-reported hypertension on treatment- controlled^†^, (%*)	Self-reported hypertension on treatment- uncontrolled^‡^, (%*)	Self-reported hypertension not on treatment, (%*)	Screen-detected hypertension^§^, (%*)	Any hypertension, (%*)
**Overall**	26315	494 (1.9)	4881 (18.5)	1154 (4.4)	13393 (50.9)	19922 (75.7)
**Age, years**						
30-59	15703	236 (1.5)	2188 (13.9)	610 (3.9)	8184 (51.5)	11218 (71.4)
60-69	7739	189 (2.3)	1893 (24.4)	405 (5.1)	3756 (49.1)	6243 (80.7)
70-79	2873	69 (2.2)	800 (27.8)	139 (4.9)	1453 (51.3)	2461 (85.7)
**Sex**						
Men	10173	145 (1.4)	1595 (15.7)	464 (4.6)	5547 (54.5)	7751 (76.2)
Women	16142	349 (2.2)	3286 (20.4)	690 (4.3)	7846 (48.6)	12171 (75.4)
**Region**						
Rural	10936	155 (1.4)	1916 (18.0)	494 (4.6)	5924 (54.3)	8489 (77.6)
Urban	15379	339 (2.2)	2965 (18.9)	660 (4.2)	7469 (48.2)	11433 (74.3)

*Standardised to age, sex and study area structure of the population, as appropriate; ^†^SBP<130mmHg and DBP<80mmHg; ^‡^SBP ≥130mmHg or DBP ≥80mmHg; ^§^No self-reported hypertension and SBP ≥130mmHg or DBP ≥80mmHg.

### 3.2 Hypertension and cardiovascular disease risk

**Fig. 1:**
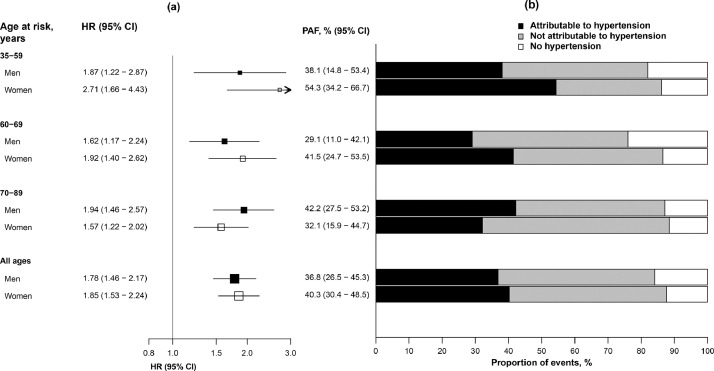
Cardiovascular deaths attributable to uncontrolled hypertension among individuals with type 2 diabetes. (a) Adjusted HR for cardiovascular death associated with uncontrolled hypertension (SBP ≥130 mmHg or DBP ≥80 mmHg) by age and sex. HRs are stratified by age-at-risk and study area and are adjusted for education, smoking, alcohol consumption, physical activity and BMI. Squares represent the HR with the area inversely proportional to the variance of the log HR, and error bars indicate the 95%CI. (b) Proportion of cardiovascular deaths attributable to uncontrolled hypertension among individuals with type 2 diabetes. Population attributable fraction (PAF) was calculated as P*([HR−1]/HR), where *P* is the proportion of individuals with type 2 diabetes who died due to cardiovascular disease with uncontrolled hypertension [27].

During median (IQR) 9 (8–10) years’ follow-up, there were 5198 incident stroke events (including 4362 IS and 469 ICH events), 1190 MCE and 4503 deaths, including 1807 cardiovascular deaths. For all CVD endpoints studied, risk increased progressively with more marked hypertension (*p* for trend ≤0.0001) (Table 3). Compared with blood pressure in the normotensive range, stage 1 hypertension (SBP 130–139 mmHg or DBP 80–89 mmHg) was associated with ~40% higher risk of ischaemic CVD (MCE: HR 1.41 [95% CI 1.24–1.61]; IS 1.35 [1.26–1.44]), increasing to almost 2.5-fold higher risk in the hypertensive crisis range (SBP ≥180 mmHg or DBP ≥120 mmHg) (MCE 2.35 [1.96–2.82]; IS 2.42 [2.19–2.68]). For ICH, stage 1 hypertension was associated with similar risk (HR 1.33 [95% CI 1.02–1.72]), but risks with more marked hypertension were higher (hypertensive crisis range: 6.02 [4.83–7.51]). Hypertension-associated risks of cardiovascular mortality were intermediate (stage 1 hypertension: HR 1.39 [95% CI 1.24–1.56]; hypertensive crisis: 3.64 [3.21–4.12]). In T2D, 10-year CVD risks increased progressively from 16% among individuals with *normal* blood pressure, to 53% in the hypertensive crisis range, compared with 6% and 40%, respectively, among individuals without diabetes (**Fig. S1**). Assuming a causal relationship, 39% of cardiovascular deaths among adults with T2D in China can be attributed to uncontrolled hypertension (Fig. 1).

**Table 3:** Associations of hypertension with major cardiovascular diseases and all-cause mortality among individuals with diabetes.

**Hypertension status**	**Major coronary event**		**Ischaemic stroke**		**Intracerebral haemorrhage**		**Total stroke**		**Cardiovascular death**		**All-cause mortality**	
	**Events**	**HR (95% CI)**	**Events**	**HR (95% CI)**	**Events**	**HR (95% CI)**	**Events**	**HR (95% CI)**	**Events**	**HR (95% CI)**	**Events**	**HR (95% CI)**
Normotensive^a^	92	1.00 (0.81–1.23)	399	1.00 (0.90–1.11)	23	1.00 (0.66–1.52)	458	1.00 (0.91–1.10)	116	1.00 (0.83–1.21)	476	1.00 (0.91–1.10)
Elevated blood pressure	101	1.14 (0.94–1.39)	445	1.30 (1.18–1.43)	32	1.35 (0.95–1.91)	512	1.29 (1.18–1.41)	135	1.16 (0.98–1.38)	415	0.89 (0.81–0.98)
Stage 1 hypertension	230	1.41 (1.24–1.61)	868	1.35 (1.26–1.44)	58	1.33 (1.02–1.72)	1002	1.34 (1.26–1.43)	291	1.39 (1.24–1.56)	824	0.98 (0.91–1.05)
Stage 2 hypertension	644	1.72 (1.59–1.86)	2262	1.71 (1.64–1.79)	270	2.77 (2.45–3.12)	2721	1.78 (1.71–1.85)	1003	2.03 (1.91–2.16)	2300	1.24 (1.18–1.29)
Hypertensive crisis	123	2.35 (1.96–2.82)	388	2.42 (2.19–2.68)	86	6.02 (4.83–7.51)	505	2.68 (2.45–2.93)	262	3.64 (3.21–4.12)	488	1.85 (1.69–2.03)
*p for trend*		*<0.0001*		*<0.0001*		*<0.0001*		*<0.0001*		*<0.0001*		*<0.0001*

a Reference group. Analyses stratified by age-at-risk, sex and study area, and adjusted for education, smoking, alcohol, physical activity and BMI. Normotensive: SBP <120 mmHg and DBP <80 mmHg; Elevated blood pressure: SBP 120–129 mmHg and DBP <80 mmHg; Stage 1 hypertension: SBP 130–139 mmHg or DBP 80–89 mmHg; Stage 2 hypertension: SBP 140–179 mmHg or DBP 90–119 mmHg; Hypertensive crisis: SBP ≥180 mmHg or DBP ≥120 mmHg; individuals who fall into two hypertension categories are designated to the higher category.

### 3.3 Usual blood pressure and cardiovascular disease risk

**Fig. 2:**
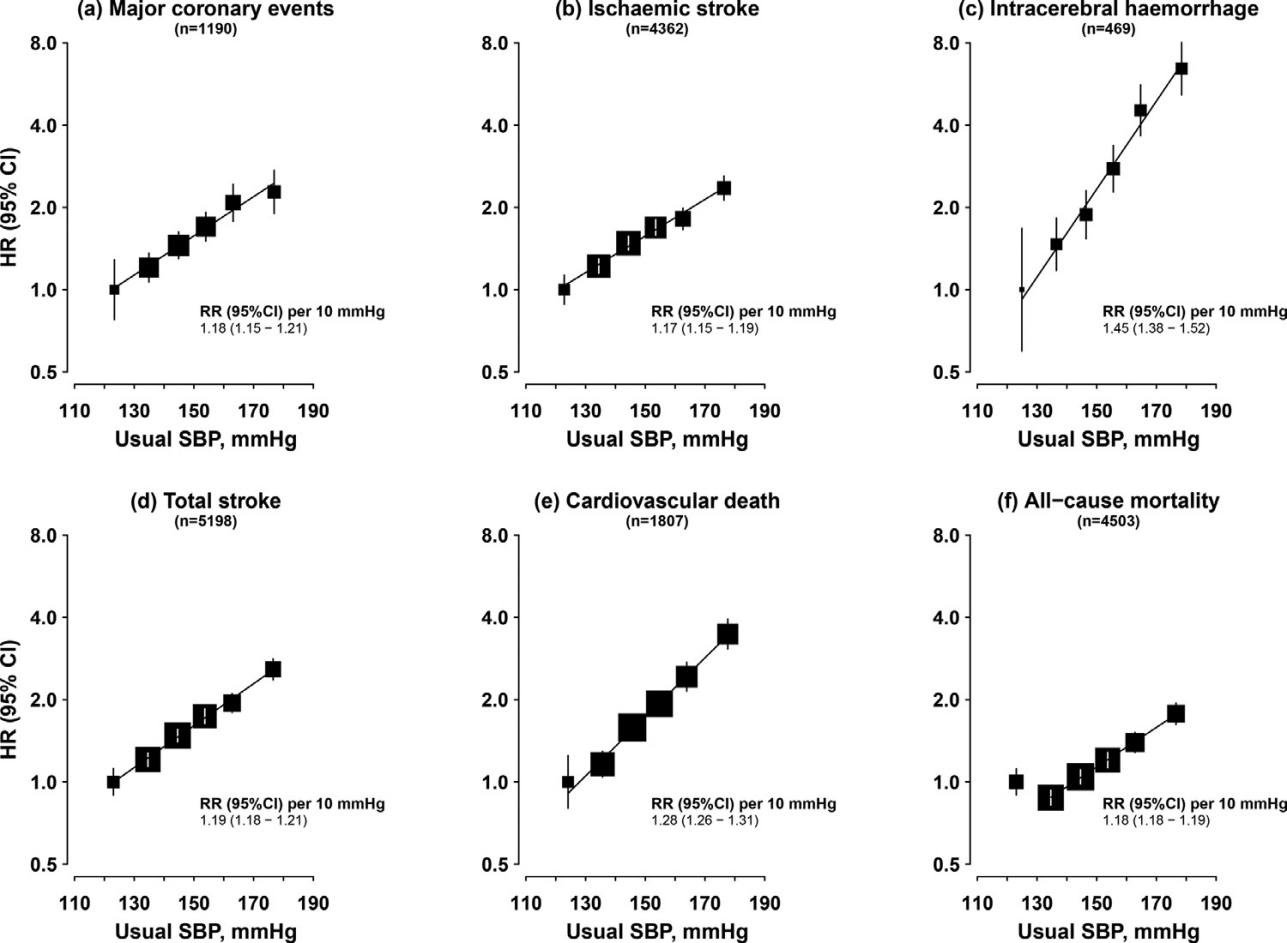
Associations of usual systolic blood pressure with major cardiovascular diseases and all-cause mortality among individuals with type 2 diabetes. Stratified by age-at-risk, sex and study area and adjusted for education, smoking, alcohol consumption, physical activity and BMI. SBP category cut-points: baseline SBP <115 mmHg, 115 to <135 mmHg, 135 to <150 mmHg, 150 to <165 mmHg, 165 to <180 mmHg, ≥180 mmHg. Squares represent the HR with area inversely proportional to the variance of the log HR, and error bars indicate the 95% CI. Adjusted HRs are plotted against mean usual SBP levels in each category.

There were strong positive log-linear associations of usual (longer-term average) SBP with risk of CVD, with no evidence of a threshold down to at least ~120 mmHg (Fig. 2). Each 10 mmHg higher usual SBP was associated with 18% (HR 1.18, 95% CI [1.15–1.21]) and 17% (1.17 [1.15–1.19]) higher risks of MCE and IS, respectively. For ICH, the association was more than twice as strong (HR 1.45 [95% CI 1.38–1.52]). The association with cardiovascular death was intermediate between these (1.28 [1.26–1.31]). There was a J-shaped association of usual SBP with all-cause mortality; risk was lowest at ~130 mmHg, with a positive log-linear association above this (HR 1.18 [95% CI 1.18–1.19] per 10 mmHg).

Usual DBP showed similar log-linear and J-shaped associations with CVD outcomes and all-cause mortality, respectively (**Fig. S2**). Additional adjustment for DBP attenuated the associations of usual SBP with CVD and with all-cause mortality by ~25–40%, but significant positive associations remained (**Fig. S3**). After additional adjustment for SBP, the associations of usual DBP with major ischaemic CVD and with all-cause mortality were fully attenuated, but a positive association remained with ICH (**Fig. S4**).

There were similar shaped associations of usual SBP with risks of major CVD and all-cause mortality across age groups (**Fig. S5**), but the associations with MCE, total stroke and cardiovascular death were stronger at younger than at older ages (*p* for trend ≤0.007) (**Fig. S6**). There were otherwise little differences in the strength of these associations across population subgroups or study areas (**Fig. S7**). There were no consistent differences in the associations of usual SBP between individuals with self-reported and screen-detected T2D (**Fig. S8**), and the associations were similar among participants with and without self-reported hypertension (**Fig. S9**). Sensitivity analyses including individuals with prior CVD (**Fig. S10**), additionally excluding individuals with a history of other chronic diseases and poor self-rated health at baseline (**Fig. S11**), or additionally adjusting for family history of CVD, diabetes and CVD medication use, or duration of diabetes, did not materially alter the findings. Compared with those without diabetes, the associations of usual SBP and DBP with CVD and with all-cause mortality among individuals with T2D were somewhat more modest (**Figs. S2 and S12**). However, the absolute risks were significantly greater at any given level of blood pressure among individuals with, than without, diabetes.

## 4 Discussion

In this large, nationwide, prospective cohort of Chinese adults with T2D but no prior CVD, hypertension was common, but poorly diagnosed and controlled. Usual blood pressure was strongly positively and log-linearly associated with risk of incident major CVD and cardiovascular mortality, with no evidence of a threshold throughout the range examined. If these associations are causal, 39% of cardiovascular deaths among adults with T2D in China could be attributed to uncontrolled hypertension.

Compared with CKB participants without diabetes, hypertension was more prevalent in T2D (~55% vs. ~75%), consistent with frequent co-occurrence of these conditions [2]. Although blood pressure lowering treatment among those with previously diagnosed hypertension was more common in T2D [19], the proportion controlled was similar irrespective of diabetes status. Blood pressure control in diabetes often requires multiple anti-hypertensive therapies [16], however, the majority of diagnosed hypertension in the present study was treated with a single agent, which may partly explain the low control rate. Moreover, ACE-inhibitors were rarely used, despite their established benefits in diabetes [16]. Nationally representative population-based estimates from China of hypertension prevalence and management in diabetes are lacking. A 2010–11 nationwide study of ~25,000 outpatients with T2D, with an average age of 63 years, reported that 60% had diagnosed hypertension, of whom 77% were treated and 17% were controlled (<130/80 mmHg) [29]. Despite this previous study focusing only on patients with established T2D receiving hospital-based care [29], these findings are consistent with those in the present study, and both highlight the need for improved hypertension detection and management among individuals with diabetes in China, underscored by the high prevalence of other CVD risk factors (e.g., smoking among men), and infrequent use of cardio-protective medications (e.g., statins).

The present study demonstrated strong positive log-linear associations of SBP with incident MCE and stroke, with no evidence of a threshold down to usual SBP levels of at least ~120 mmHg. This is broadly consistent with previous investigations showing continuous positive associations with IHD and stroke extending to baseline and usual SBP levels of 110–120 mmHg [6,30] and ~115–120 mmHg [3,^7^,31], respectively. Previous studies have repeatedly observed stronger associations of blood pressure with stroke than with IHD [6,^7^,30]. For example, observational analysis of UK Prospective Diabetes Study (UKPDS) data, including 3642 individuals with newly-diagnosed T2D, found 12% (95% CI 7–16%) and 19% (95% CI 14–24%) lower risk of MI and stroke, respectively, per 10 mmHg reduction in mean SBP [7]. In contrast, although the association with IHD (HR 1.18) in CKB was consistent with other studies [3,7], there was a similar association with total stroke (1.19). A retrospective cohort study, based on routine healthcare data for ~180,000 patients with diagnosed T2D in Hong Kong, also found similar strengths of association of usual SBP with IHD (1.10 [95% CI 1.01–1.12] per 10 mmHg higher) and total stroke (1.11 [1.09–1.13]), albeit weaker than those observed in the present study, possibly reflecting less robust CVD phenotyping [31]. Previous investigations of the associations of blood pressure with stroke types in diabetes have been constrained by small numbers of stroke cases [3], and CKB provides the most reliable risk estimates to-date, demonstrating a stronger association with ICH than with IS. This is consistent with analyses within the overall CKB cohort (HR 1.68 [95% CI 1.65–1.71] vs. 1.30 [1.29–1.31] per 10 mmHg higher usual SBP) [32], but differs from findings from a general population, individual participant data meta-analysis of one million participants, which showed no heterogeneity in the association of usual SBP with IS and haemorrhagic stroke mortality [4]. The comparable strengths of association with MCE and IS in the present study might reflect the frequent use of neuroimaging in China, detecting less severe stroke events, while the stronger association with ICH may partly reflect its high case fatality rate [33].

Controversy persists regarding optimal blood pressure targets in diabetes [13–16]. Although the observational nature of the present analyses precludes causal inferences, they provide support for more intensive treatment targets (i.e., <130/80 mmHg) for CVD prevention, consistent with ACC/AHA^13^ and Chinese Diabetes Society guidelines [14]. Furthermore, the consistency of the observed associations irrespective of prior CVD suggests this is relevant to both primary and secondary CVD prevention. Moreover, the stronger association with ICH further serves to highlight the potential value of such targets among populations (e.g., in China) with high stroke incidence and mortality [34].

A positive log-linear association between blood pressure and cardiovascular mortality was observed in the present study, contrasting with the apparent J-shaped association with all-cause mortality. Analysis of data from the Swedish national diabetes register, including ~190,000 individuals with T2D and without previous CVD or other major diseases, similarly showed significantly higher all-cause mortality risk among individuals with baseline SBP levels of 110–119 mmHg (HR 1.28 [95% CI 1.15–1.42]) than 120–129 mmHg (1.05 [0.97–1.13]) and 130–139 mmHg (1.00), with a positive association above this [6]. In contrast, and consistent with general population studies [4,32], the UKPDS analyses suggested no flattening or higher risk at low SBP [7]. Attempts to address reverse causality in CKB did not alter the observed associations. However, deaths in the lowest SBP category were among individuals with low BMI and high rates of smoking and poor self-rated health, suggestive of residual reverse causality. The apparent higher all-cause mortality risk at lower blood pressure levels may also reflect susceptible subgroups. This is supported partly by trial evidence, suggesting blood pressure lowering may be less beneficial among individuals with diabetes who have lower baseline blood pressure levels [35,36]. However, the observed association with all-cause mortality risk highlights the need for caution in adopting more intensive blood pressure treatment targets and the value of personalised approaches in T2D.

The present study has several strengths, including the large numbers of well-phenotyped CVD cases, and, in particular, neuroimaging-confirmed (~90%) stroke cases, permitting the largest investigation to-date of the association of blood pressure with stroke types in T2D. In combination with reliable blood pressure measurements, including repeat measurements among a subset of participants, this provides robust estimates of the association of average long-term blood pressure with risk of major CVD. However, our study has certain limitations. Firstly, it was not possible to adjust for blood lipid levels, HbA1c levels or renal function in the present analyses, which may have resulted in residual confounding of the association of blood pressure with cardiovascular disease. Secondly, although attempts were made to eliminate reverse causality, individuals with prior heart failure could not be excluded, which may have influenced the mortality findings [37]. Thirdly, we were unable to identify participants with white coat or masked hypertension. Adjustment for regression dilution bias may in part have accounted for these phenomena, however, residual under- or over-estimation of blood pressure associated CVD risks may still exist. Fourthly, screen-detected diabetes was likely under-diagnosed due to reliance on glucose (without HbA1c) levels for diagnosis, and limited phenotyping data available to differentiate between diabetes types may have resulted in inclusion of some participants with diabetes types other than T2D. However, such misclassification would not be expected to impact significantly on the presented risk estimates. Finally, CKB is not designed to be nationally representative; given the study population size and diversity, this would not be expected to bias risk estimates, but may impact on estimated disease burden.

In conclusion, the present study provides important evidence of strong, continuous, positive risk associations of blood pressure with CVD among adults with T2D in China, and highlights the need for effective blood pressure control. Despite these continuous associations, treatment thresholds and targets in hypertension are valuable in clinical management and the present findings support lower advocated thresholds (<130/80 mmHg) for CVD prevention. They also provide reliable evidence of the need for improved hypertension detection and management among adults with T2D in China, which, given demonstrated causal associations of blood pressure with CVD [38], would be expected to have a substantial impact on the diabetes-associated CVD burden in Chinese adults.

## Author contributions

FB, ZC, JH and LL had full access to the data. All authors were involved in study design, conduct, long-term follow-up, analysis of data, interpretation, or writing the report.

## Declaration of Interests

We declare that we have no conflict of interest.

## Appendix Supplementary materials

Image, application 1
