# Supplementary material for: Blood pressure and cardiovascular diseases in Chinese adults with type 2 diabetes: A prospective cohort study
Source: Lancet Reg Health West Pac. 2021 Jan 23;7:100085. doi: 10.1016/j.lanwpc.2020.100085 (PMC8315364; doi:10.1016/j.lanwpc.2020.100085)

This page is intentionally left blank

Supplementary appendix, Table of Contents

Page 3: China Kadoorie Biobank collaborative group and members

Page 4: Table S1. Prevalence, treatment and control of hypertension among individuals with self-reported and screen-detected type 2 diabetes

Page 5: Table S2. Prevalence, treatment and control of hypertension among individuals with type 2 diabetes

Page 6. Figure S1. 10-year cardiovascular disease risk by hypertension status among individuals with and without type 2 diabetes

Page 7: Figure S2. Associations of usual DBP with major cardiovascular diseases and all-cause mortality by diabetes status

Page 8: Figure S3. Associations of usual SBP with cardiovascular diseases and all-cause mortality among individuals with type 2 diabetes, additionally adjusted for DBP

Page 9: Figure S4. Associations of usual DBP with cardiovascular diseases and all-cause mortality among individuals with type 2 diabetes, additionally adjusted for SBP

Page 10: Figure S5. Associations of usual SBP with cardiovascular diseases and all-cause mortality among individuals with type 2 diabetes by age

Page 11: Figure S6. Risks of major coronary event, total stroke and cardiovascular death per 10 mmHg higher usual SBP among individuals with type 2 diabetes

Page 12: Figure S7. Risks of cardiovascular death per 10 mmHg higher usual SBP among individuals with type 2 diabetes by study area

Page 13: Figure S8. Associations of usual SBP with major cardiovascular diseases and all-cause mortality among individuals with self-reported and screen-detected type 2 diabetes

Page 14: Figure S9. Associations of usual SBP with major cardiovascular diseases and all-cause mortality among individuals with type 2 diabetes by self-reported hypertension status

Page 15: Figure S10. Associations of usual SBP with major cardiovascular diseases and all-cause mortality among individuals with type 2 diabetes, not excluding individuals with prior cardiovascular diseases

Page 16: Figure S11. Associations of usual SBP with all-cause mortality among individuals with type 2 diabetes excluding individuals with (a) prior chronic diseases and (b) prior chronic diseases and poor self-reported health

Page 17: Figure S12. Associations of usual SBP with major cardiovascular diseases and all-cause mortality by diabetes status

China Kadoorie Biobank collaborative group and members

International Steering Committee: Junshi Chen, Zhengming Chen (PI), Robert Clarke, Rory Collins, Yu Guo, Liming Li (PI), Jun Lv, Richard Peto, Robin Walters.

International Co-ordinating Centre, Oxford: Daniel Avery, Derrick Bennett, Ruth Boxall, Fiona Bragg, Yumei Chang, Yiping Chen, Zhengming Chen, Robert Clarke, Huaidong Du, Simon Gilbert, Alex Hacker, Michael Holmes, Andri Iona, Christiana Kartsonaki, Rene Kerosi, Sarah Lewington, Garry Lancaster, Kuang Lin, John McDonnell, Iona Millwood, Qunhua Nie, Paul Ryder, Sam Sansome, Dan Schmidt, Paul Sherliker, Rajani Sohoni, Becky Stevens, Iain Turnbull, Robin Walters, Lin Wang, Neil Wright, Ling Yang, Xiaoming Yang.

National Co-ordinating Centre, Beijing: Ge Chen, Yu Guo, Xiao Han, Can Hou, Jun Lv, Pei Pei, Shuzhen Qu, Yunlong Tan, Canqing Yu.

10 Regional Co-ordinating Centres: Qingdao Qingdao CDC: Zengchang Pang, Ruqin Gao, Shaojie Wang, Yongmei Liu, Ranran Du, Yajing Zang, Liang Cheng, Xiaocao Tian, Hua Zhang. Licang CDC: Silu Lv, Junzheng Wang, Wei Hou. **Heilongjiang** Provincial CDC: Jiyuan Yin, Ge Jiang, Xue Zhou. Nangang CDC: Liqiu Yang, Hui He, Bo Yu, Yanjie Li, Huaiyi Mu, Qinai Xu, Meiling Dou, Jiaojiao Ren. **Hainan** Provincial CDC: Shanqing Wang, Ximin Hu, Hongmei Wang, Jinyan Chen, Yan Fu, Zhenwang Fu, Xiaohuan Wang. Meilan CDC: Min Weng, Xiangyang Zheng, Yilei Li, Huimei Li,Yanjun Wang. **Jiangsu** Provincial CDC: Ming Wu, Jinyi Zhou, Ran Tao, Jie Yang. Suzhou CDC: Chuanming Ni, Jun Zhang, Yihe Hu, Yan Lu, , Liangcai Ma, Aiyu Tang, Shuo Zhang, Jianrong Jin, Jingchao Liu. **Guangxi** Provincial CDC: Zhenzhu Tang, Naying Chen, Ying Huang. Liuzhou CDC: Mingqiang Li, Jinhuai Meng, Rong Pan, Qilian Jiang, Weiyuan Zhang, Yun Liu, Liuping Wei, Liyuan Zhou, Ningyu Chen, Hairong Guan. **Sichuan** Provincial CDC: Xianping Wu, Ningmei Zhang, Xiaofang Chen, Xuefeng Tang. Pengzhou CDC: Guojin Luo, Jianguo Li, Xiaofang Chen, Xunfu Zhong, Jiaqiu Liu, Qiang Sun. **Gansu** Provincial CDC: Pengfei Ge, Xiaolan Ren, Caixia Dong. Maiji CDC: Hui Zhang, Enke Mao, Xiaoping Wang, Tao Wang, Xi Zhang. **Henan** Provincial CDC: Ding Zhang, Gang Zhou, Shixian Feng, Liang Chang, Lei Fan. Huixian CDC: Yulian Gao, Tianyou He, Huarong Sun, Pan He, Chen Hu, Qiannan Lv, Xukui Zhang. **Zhejiang** Provincial CDC: Min Yu, Ruying Hu, Hao Wang. Tongxiang CDC: Yijian Qian, Chunmei Wang, Kaixue Xie, Lingli Chen, Yidan Zhang, Dongxia Pan. **Hunan** Provincial CDC: Yuelong Huang, Biyun Chen, Li Yin, Donghui Jin, Huilin Liu, Zhongxi Fu, Qiaohua Xu. Liuyang CDC: Xin Xu, Hao Zhang, Youping Xiong, Huajun Long, Xianzhi Li, Libo Zhang, Zhe Qiu.

Table S1. Prevalence, treatment and control of hypertension among individuals with self-reported and screen-detected type 2 diabetes

Characteristic	No. of participants	Hypertension				
		Self-reported, (%*)			Screen-detected§, (%*)	Any, (%*)
		On treatment		Not on treatment		
		Controlled†	Uncontrolled‡			
		**Self-reported diabetes**				
**Overall**	13219	352 (2.7)	3092 (23.4)	709 (5.4)	5856 (44.3)	10009 (75.7)
**Age, years**						
30-59	7222	159 (2.3)	1300 (18.1)	359 (4.9)	3263 (44.3)	5081 (70.4)
60-69	4387	134 (2.9)	1254 (28.2)	261 (5.9)	1899 (44.0)	3548 (80.9)
70-79	1610	59 (3.3)	538 (33.6)	89 (5.6)	694 (43.5)	1380 (85.7)
**Sex**						
Men	5005	112 (2.2)	961 (19.2)	278 (5.6)	2392 (47.8)	3743 (74.8)
Women	8214	240 (2.9)	2131 (25.9)	431 (5.2)	3464 (42.2)	6266 (76.3)
**Region**						
Rural	5036	101 (2.1)	1185 (24.3)	278 (5.6)	2384 (47.3)	3948 (78.4)
Urban	8183	251 (3.1)	1907 (22.9)	431 (5.2)	3472 (42.2)	6061 (74.1)
		**Screen-detected diabetes**				
**Overall**	13096	142 (1.1)	1789 (13.7)	445 (3.4)	7537 (57.6)	9913 (75.7)
**Age, years**						
30-59	8481	77 (0.9)	888 (10.4)	251 (2.9)	4921 (57.6)	6137 (72.4)
60-69	3352	55 (1.6)	639 (19.3)	144 (4.1)	1857 (55.8)	2695 (80.4)
70-79	1263	10 (0.7)	262 (20.8)	50 (4.1)	759 (60.6)	1081 (85.6)
**Sex**						
Men	5168	33 (0.6)	634 (12.3)	186 (3.6)	3155 (61.0)	4008 (77.6)
Women	7928	109 (1.4)	1155 (14.6)	259 (3.3)	4382 (55.3)	5905 (74.5)
**Region**						
Rural	5900	54 (0.9)	731 (12.5)	216 (3.7)	3540 (60.3)	4541 (77.0)
Urban	7196	88 (1.2)	1058 (14.6)	229 (3.1)	3997 (54.8)	5372 (74.7)

*Standardised to age, sex and study area structure of the population; †SBP <130mmHg and DBP <80mmHg; ‡SBP ≥130mmHg or DBP ≥80mmHg; §No self-reported hypertension and SBP ≥130mmHg or DBP ≥80mmHg.

Table S2.Prevalence, treatment and control of hypertension among individuals with type 2 diabetes

Characteristic	No. of participants	Hypertension				
		Self-reported, (%*)			Screen-detected§, (%*)	Any, (%*)
		Controlled†	On treatment Controlled†	Not on treatment		
**Overall**	26315	1308 (5.0)	4067 (15.5)	1154 (4.4)	8235 (31.3)	14764 (56.1)
**Age, years**						
30-59	15703	650 (4.2)	1774 (11.2)	610 (3.9)	4593 (28.7)	7627 (48.6)
60-69	7739	486 (6.1)	1596 (20.6)	405 (5.1)	2591 (34.0)	5078 (65.6)
70-79	2873	172 (5.7)	697 (24.3)	139 (4.9)	1051 (37.5)	2059 (71.7)
**Sex**						
Men	10173	439 (4.3)	1301 (12.8)	464 (4.6)	3332 (32.8)	5536 (54.4)
Women	16142	869 (5.4)	2766 (17.1)	690 (4.3)	4903 (30.4)	9228 (57.2)
**Region**						
Rural	10936	435 (4.0)	1636 (15.0)	494 (4.5)	3690 (33.7)	6255 (57.2)
Urban	15379	873 (5.7)	2431 (15.8)	660 (4.3)	4545 (29.6)	8509 (55.3)

*Standardised to age, sex and study area structure of the population; †SBP <140mmHg and DBP <90mmHg; ‡SBP ≥140mmHg or DBP ≥90mmHg; §No self-reported hypertension and SBP ≥140mmHg or DBP ≥90mmHg.

Figure S1. 10-year cardiovascular disease risk by hypertension status among individuals with and without type 2 diabetes

Adjusted for age, sex, study area, educational attainment, smoking, alcohol consumption, physical activity and BMI

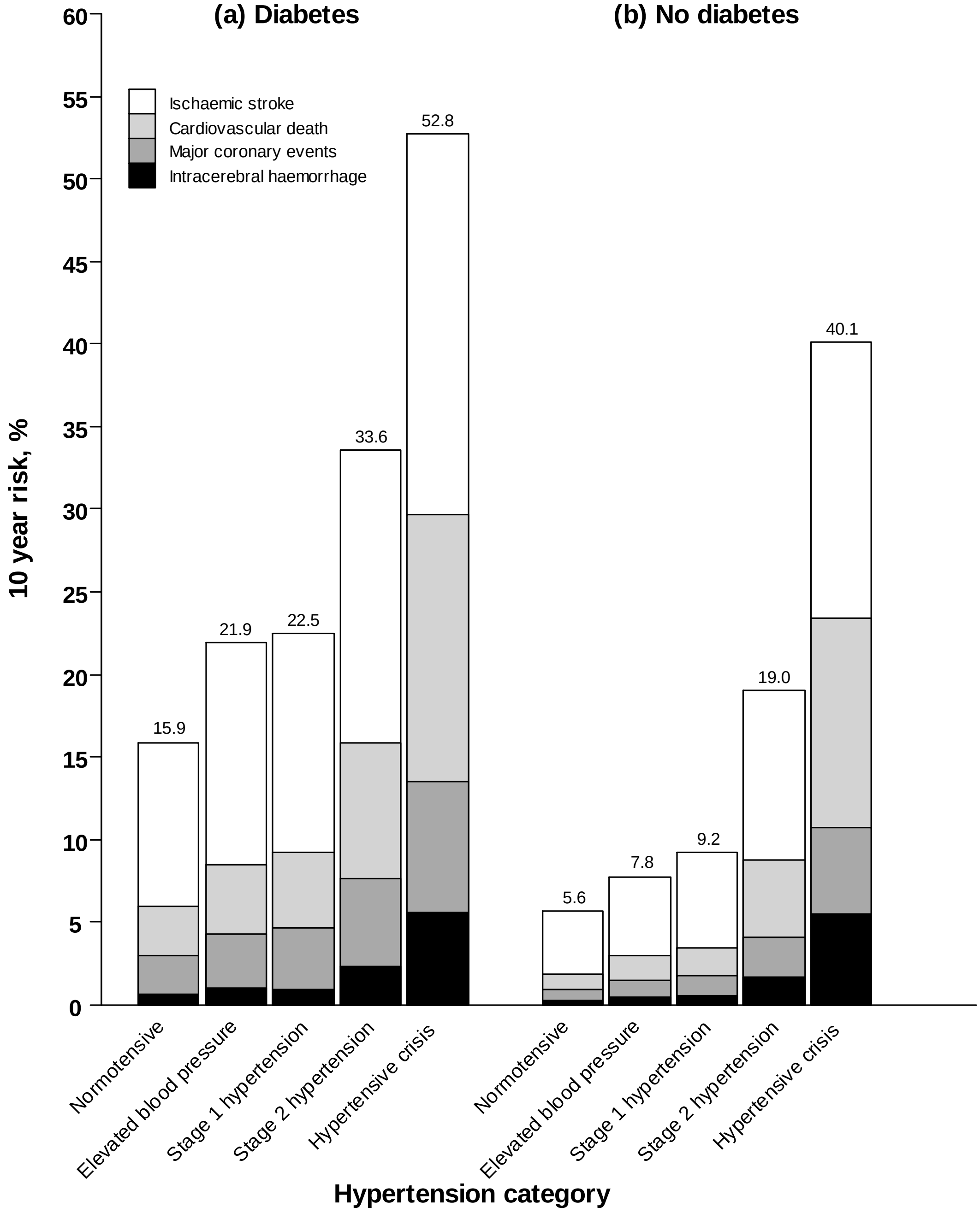

Figure S2. Associations of usual DBP with major cardiovascular diseases and all-cause mortality by diabetes status

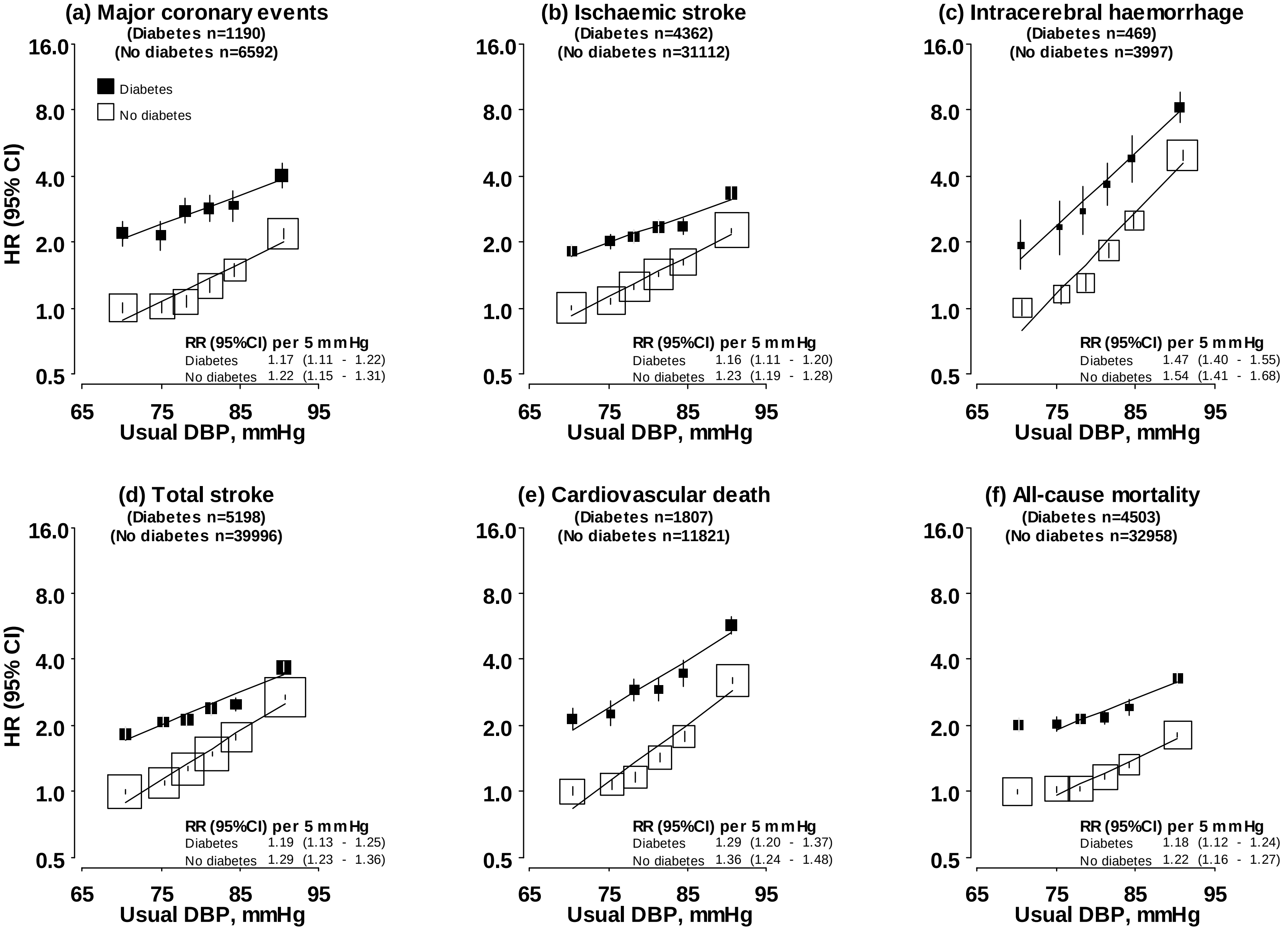

Figure S3. Associations of usual SBP with cardiovascular diseases and all-cause mortality among individuals with type 2 diabetes, additionally adjusted for DBP

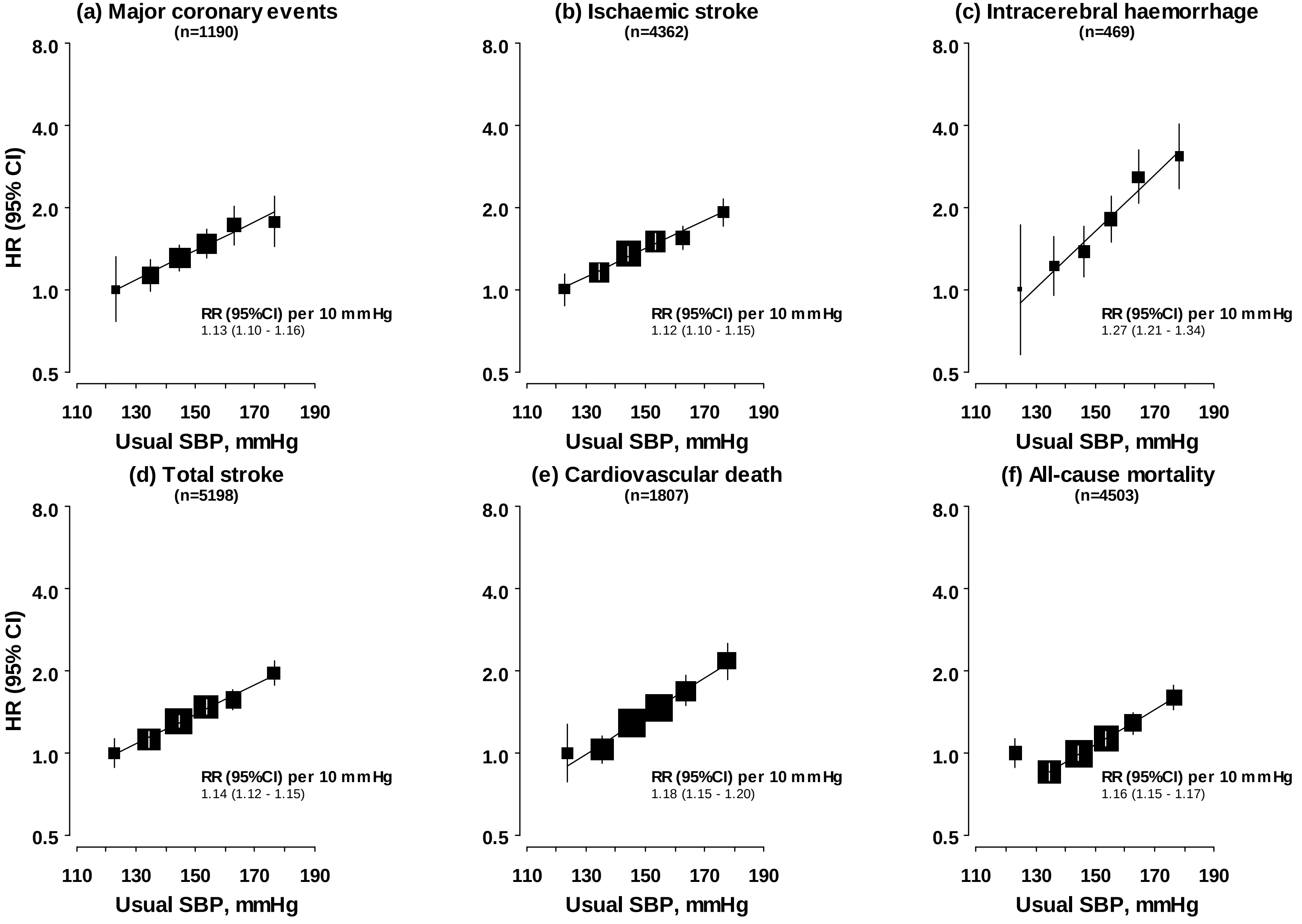

Figure S4. Associations of usual DBP with cardiovascular diseases and all-cause mortality among individuals with type 2 diabetes, additionally adjusted for SBP

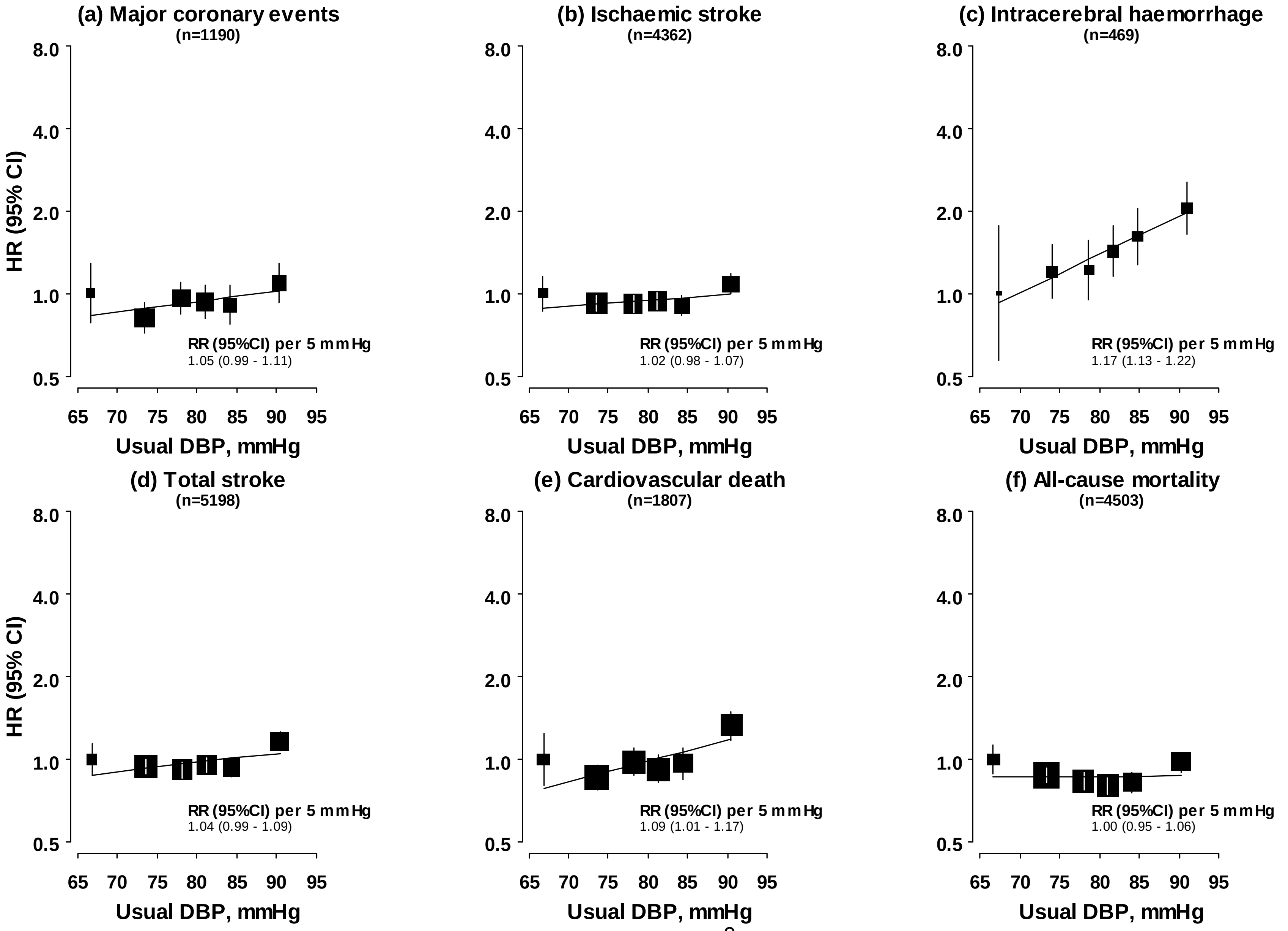

Figure S5. Associations of usual SBP with cardiovascular diseases and all-cause mortality among individuals with type 2 diabetes by age

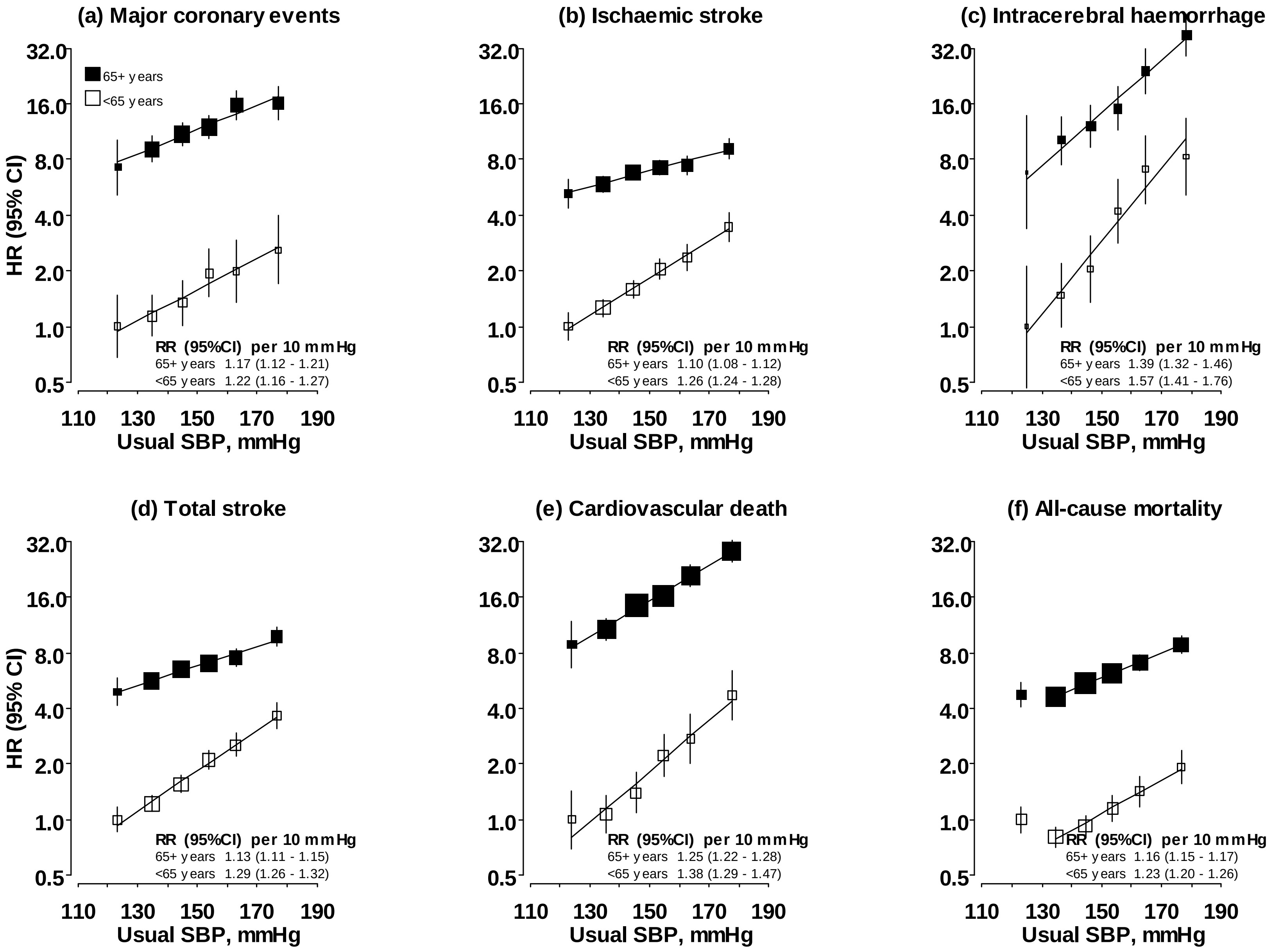

Figure S6. Risks of major coronary event, total stroke and cardiovascular death per 10 mmHg higher usual SBP among individuals with type 2 diabetes

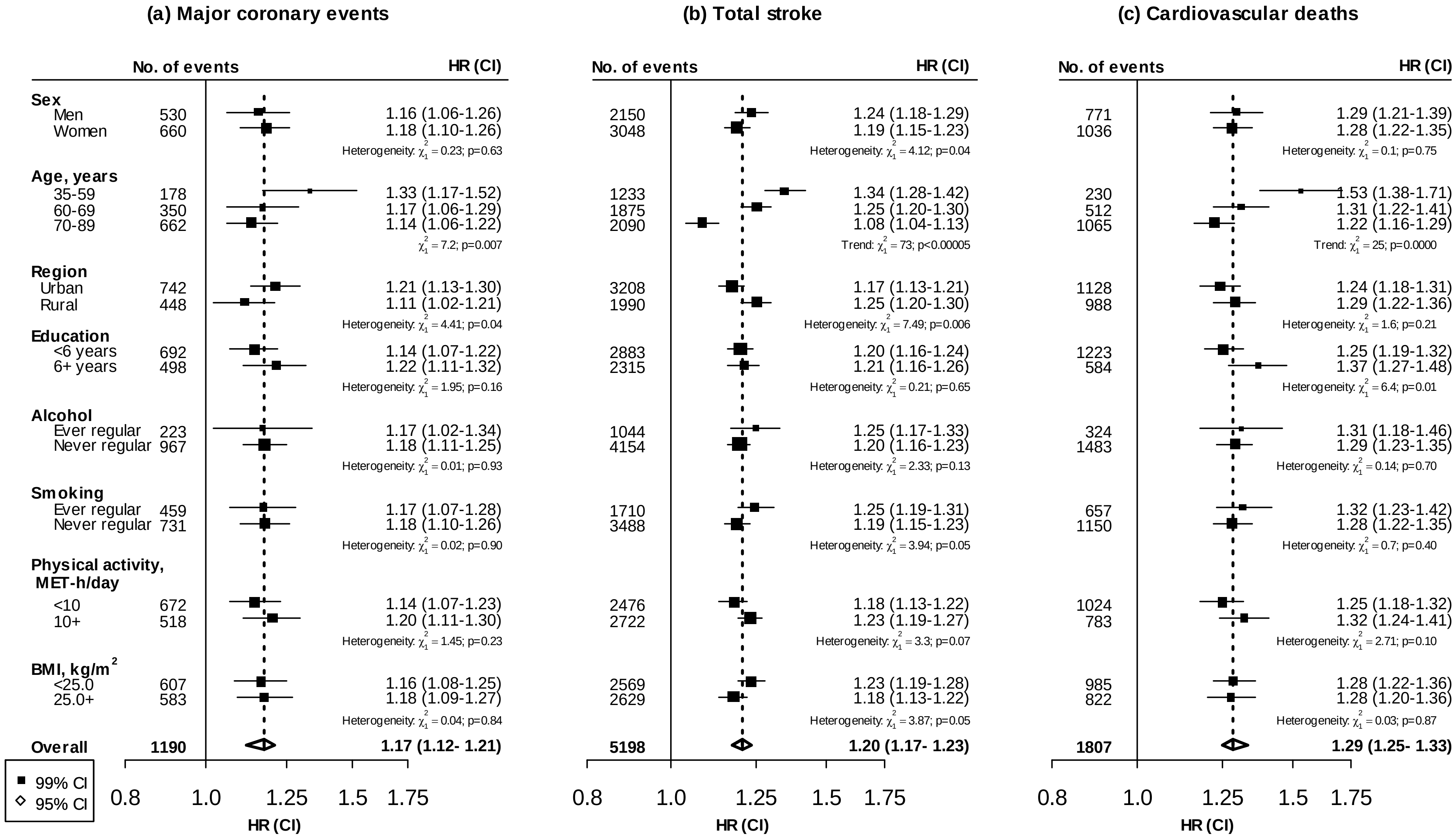

Figure S7. Risks of cardiovascular death per 10 mmHg higher usual SBP among individuals with type 2 diabetes by study area

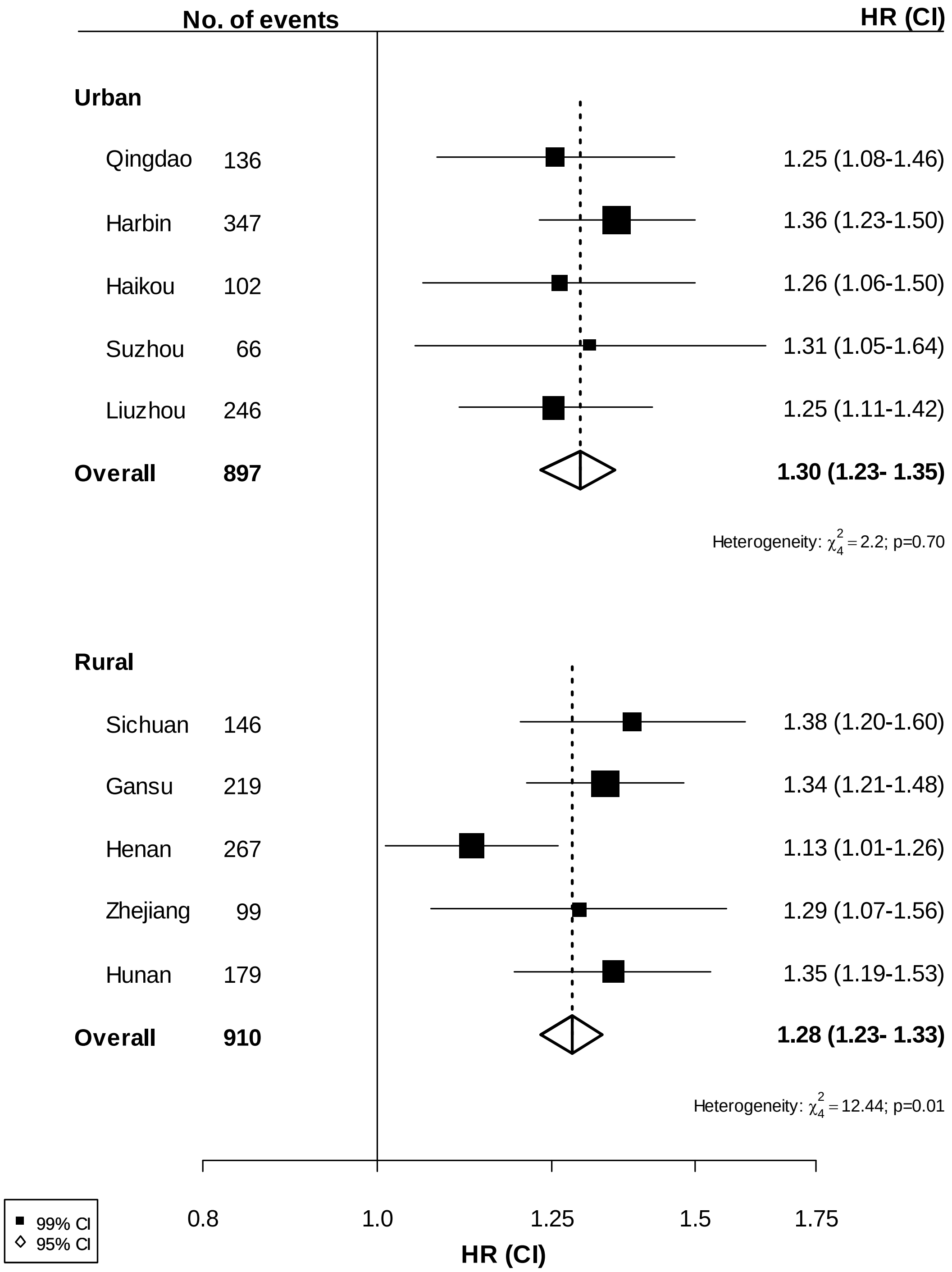

Figure S8. Associations of usual SBP with major cardiovascular diseases and all-cause mortality among individuals with self-reported and screen-detected type 2 diabetes

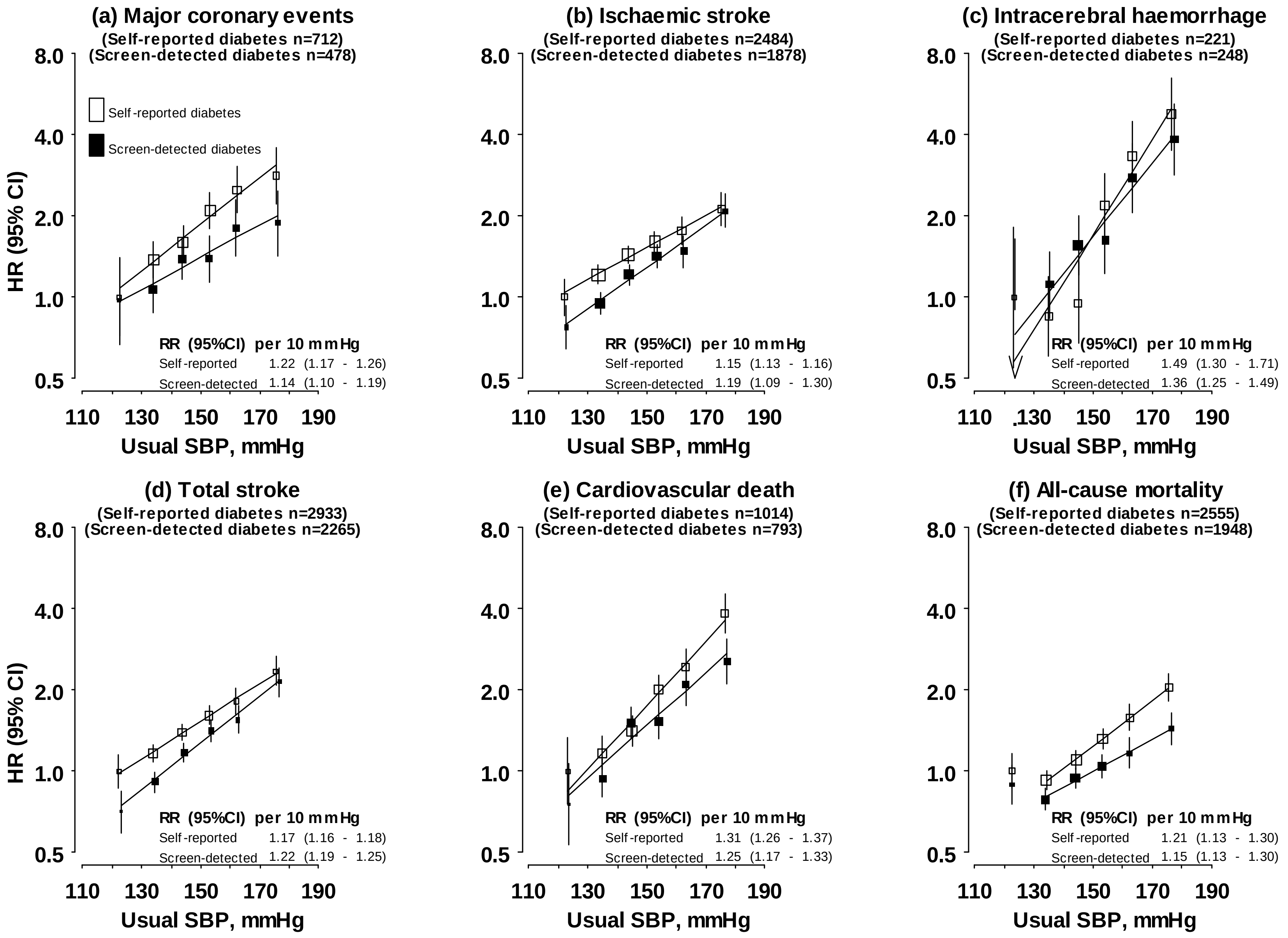

Figure S9. Associations of usual SBP with major cardiovascular diseases and all-cause mortality among individuals with type 2 diabetes by self-reported hypertension status

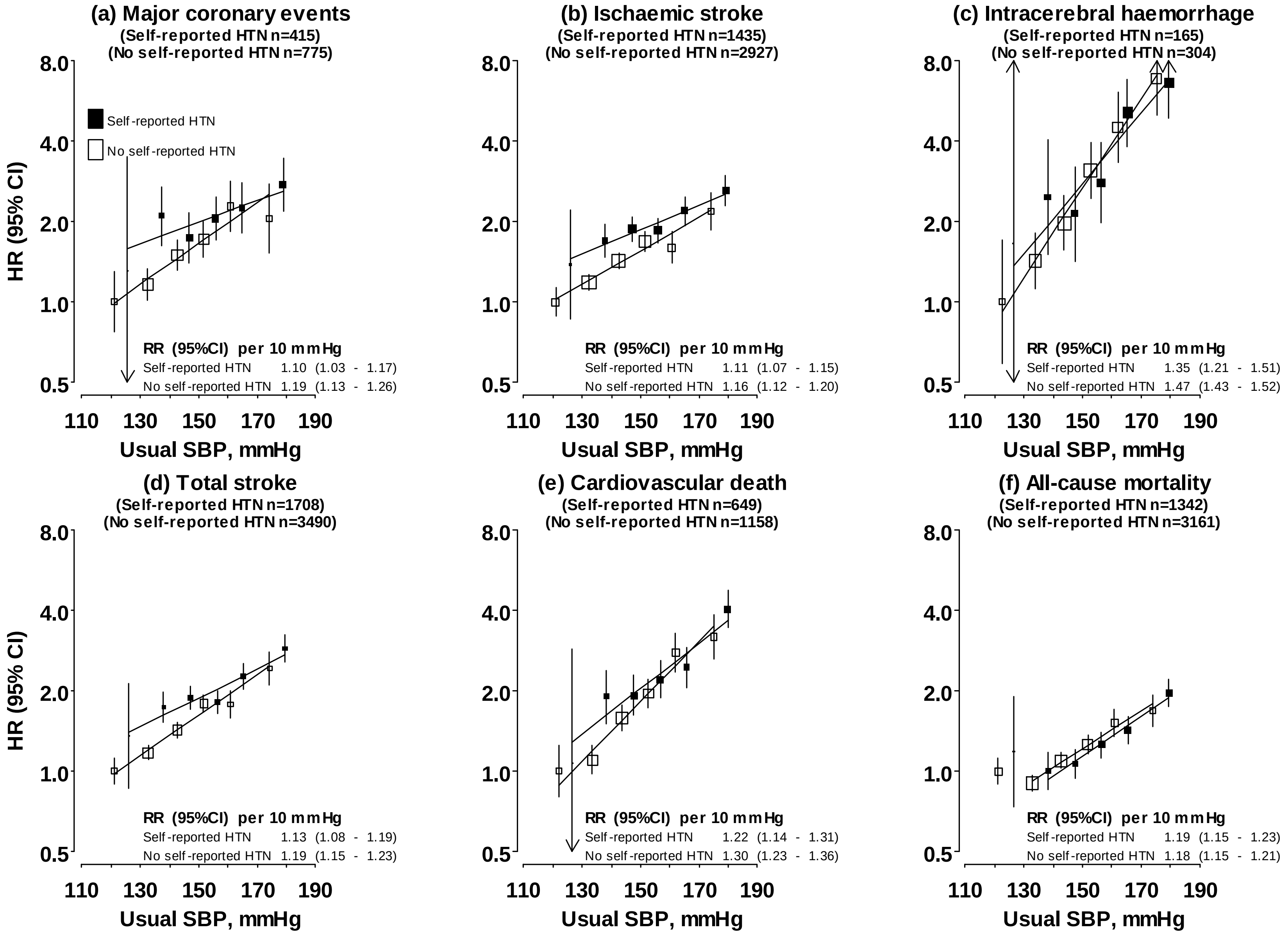

Figure S10. Associations of usual SBP with major cardiovascular diseases and all-cause mortality among individuals with type 2 diabetes, not excluding individuals with prior cardiovascular diseases

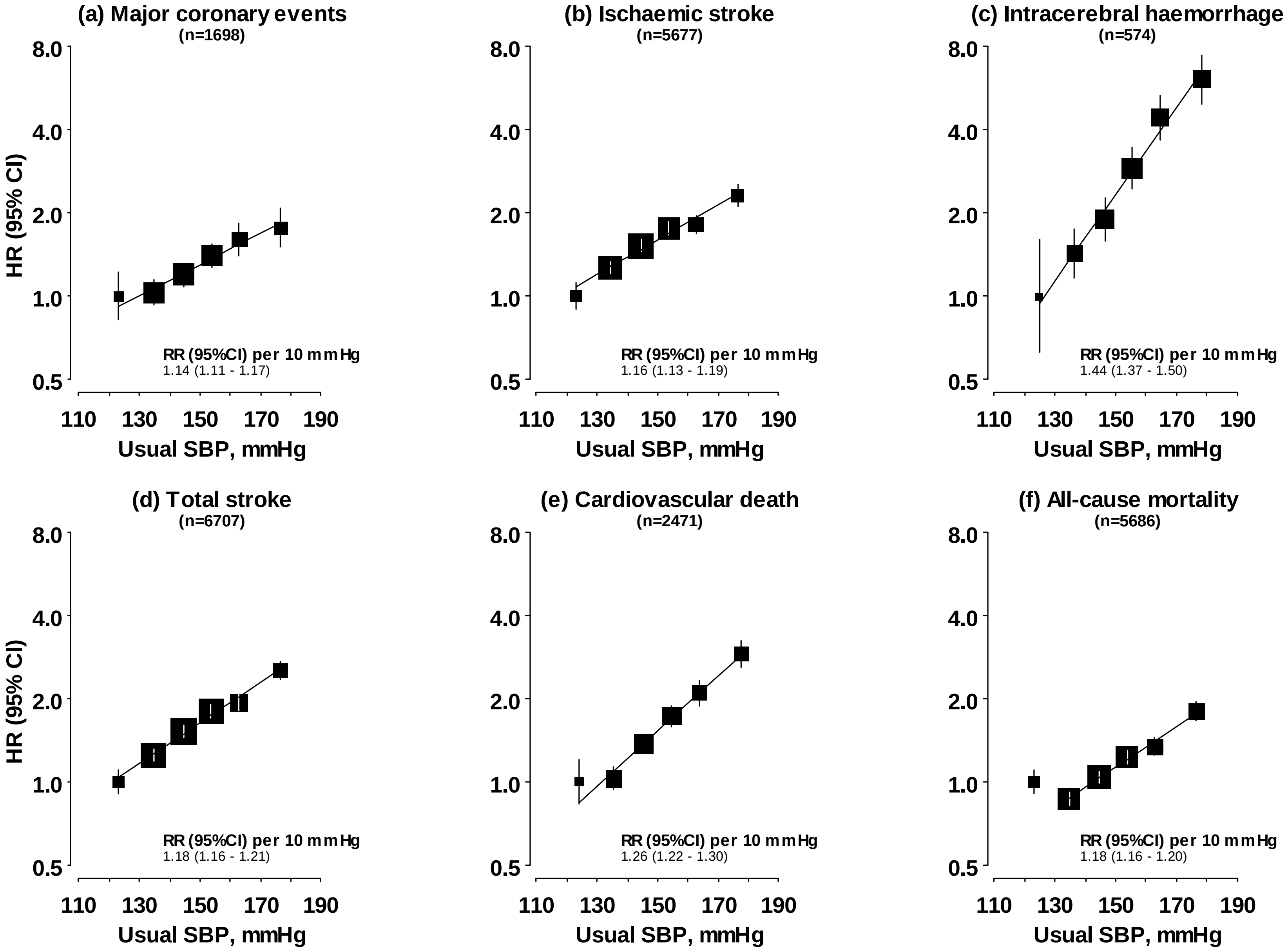

Figure S11. Associations of usual SBP with all-cause mortality among individuals with type 2 diabetes excluding individuals with (a) prior chronic diseases and (b) prior chronic diseases and poor self-reported health

Prior chronic diseases: rheumatic heart disease, chronic obstructive pulmonary disease, chronic liver disease and chronic renal disease

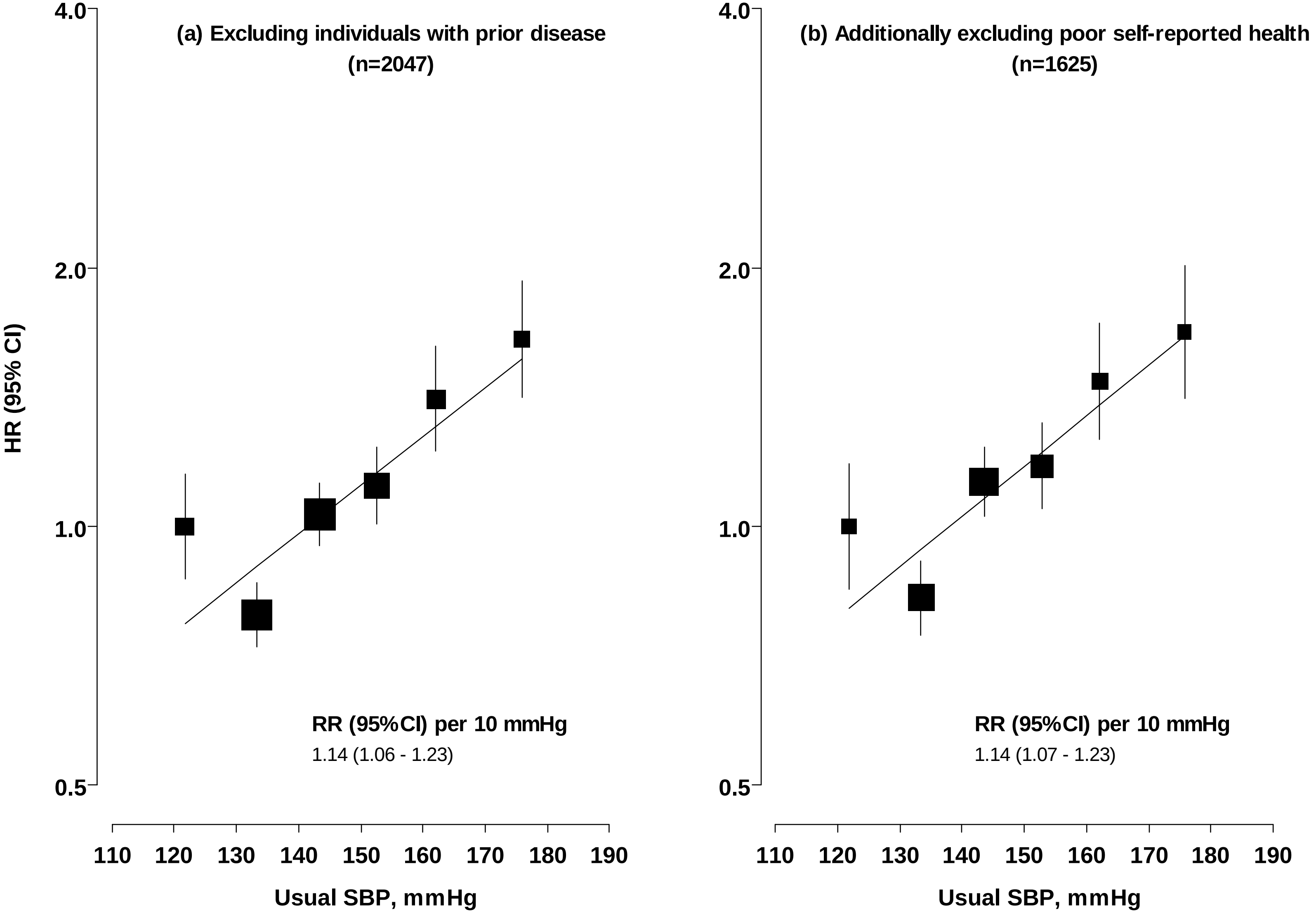

Figure S12. Associations of usual SBP with major cardiovascular diseases and all-cause mortality by diabetes status

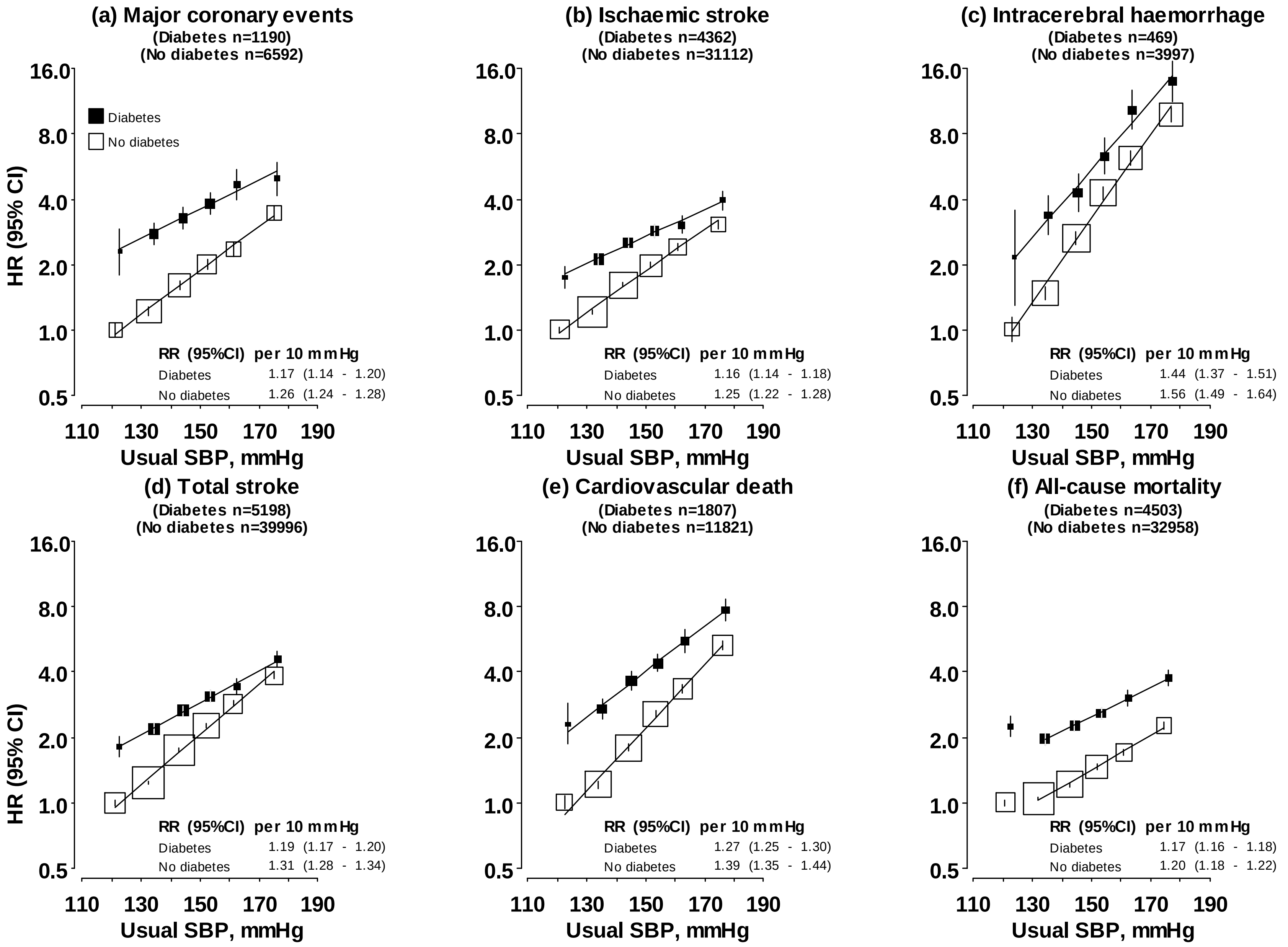

Supplement: Supplementary file 1 [file mmc1.pdf]
